# Cyclic Behavior of Low Rise Concrete Shear Walls Containing Recycled Coarse and Fine Aggregates

**DOI:** 10.3390/ma10121400

**Published:** 2017-12-07

**Authors:** Qiyun Qiao, Wanlin Cao, Zhiwei Qian, Xiangyu Li, Wenwen Zhang, Wenchao Liu

**Affiliations:** 1 College of Architecture and Civil Engineering, Beijing University of Technology, Beijing 100124, China; qiaoqiyun@bjut.edu.cn (Q.Q.); xiangyuli94@163.com (X.L.); wwzhang@emails.bjut.edu.cn (W.Z.); liuwenchao@bjut.edu.cn (W.L.); 2 Faculty of Civil Engineering and Geosciences, Delft University of Technology, 2628 CN Delft, The Netherlands

**Keywords:** low rise shear wall, recycled coarse aggregates, recycled fine aggregates, cyclic behavior, strength evaluation

## Abstract

In this study, the cyclic behaviors of low rise concrete shear walls using recycled coarse or fine aggregates were investigated. Eight low rise Recycled Aggregates Concrete (RAC) shear wall specimens were designed and tested under a cyclic loading. The following parameters were varied: replacement percentages of recycled coarse or fine aggregates, reinforcement ratio, axial force ratio and X-shaped rebars brace. The failure characteristics, hysteretic behavior, strength and deformation capacity, strain characteristics and stiffness were studied. Test results showed that the using of the Recycled Coarse Aggregates (RCA) and its replacement ratio had almost no influence on the mechanical behavior of the shear wall; however, the using of Recycled Fine Aggregates (RFA) had a certain influence on the ductility of the shear wall. When the reinforcement ratio increased, the strength and ductility also increased. By increasing the axial force ratio, the strength increased but the ductility decreased significantly. The encased brace had a significant effect on enhancing the RAC shear walls. The experimental maximum strengths were evaluated with existing design codes, it was indicated that the strength evaluation of the low rise RAC shear walls can follow the existing design codes of the conventional concrete shear walls.

## 1. Introduction

With the development of urban construction and urbanization, concrete structures have been widely and rapidly constructed, especially in some developing countries. As a result, the shortage of resources, such as the nature coarse or fine aggregates is becoming an urgent matter. Moreover, a huge amount of the construction and demolition wastes are generated due to the limitation of the structure life span or the natural hazards. Among these construction wastes, the concrete construction waste causes extremely serious environment problems. Concrete industry is responsible of the most significant contribution to the global warming due to the large amount of substances with environmental impacts produced during its entire life cycle (production process, construction, maintenance, dismantlement, and scrapping). The most important issue characterizing the concrete industry is related to the constant growth of consumption of natural aggregates. Every year, European Union generates more than 450 million tons of construction and demolition waste [1] and China produces about 200 million tons of waste concrete [2]. Such huge quantities of waste have major impact on the environment. As an effective solution to the construction and demolition wastes, Recycled Aggregates Concrete (RAC) is attracting more research and engineering activities in terms of advantages from a social, economic, and sustainable point of view. RAC can save landfill space, preserve natural resources and reduce carbon footprint. RAC is produced by crushing, cleaning and grading the construction and demolition concrete waste, forming the recycled aggregates in a certain proportion, and substituting the natural aggregates partially or entirely. The application of RAC is an important method to promote the recycling of construction waste. Furthermore, the RAC is expected to solve the increasingly serious shortage of natural aggregates, and to protect the resources and environment. 

The performance of RAC has been studied in the past decades. These studies were mainly focused on the material behavior, the application on the structures and life cycle assessment. A huge amount of studies on the RAC material have been carried out [3,4,5,6,7,8,9,10]. Also, many studies have been carried out on the RAC structures [11,12,13,14,15]. These studies indicated that, in general, the mechanical behavior (i.e., strength, ductility and elastic modulus) of the RAC is slightly inferior than the Natural Aggregates Concrete (NAC), the main reason is that most of the adverse effects (due to the presence of RCA) are because of higher water absorption and lower density of the Recycled Aggregate Concrete (RAC) due to the presence of the residual mortar on the surfaces of RAC particles [16], however, it is possible and feasible to use the RAC material in the constructions. Also, in recent years, many studies have been carried out on the standard protocol of life cycle assessment [17,18].

In the seismic design, low rise Reinforced Concrete (RC) shear walls, with height-to-width ratio less than two, are widely used for earthquake resistance in structures. Many studies have demonstrated the excellent seismic behavior of the low rise RC shear walls [19,20,21,22,23]. The development of low rise RAC shear walls has become a significant demand for the recycling of building materials and construction waste. However, studies on the RAC shear walls are limited compared with the studies on RAC columns or RAC beams. Peng et al. [24] investigated the influence of the axial load level and the amount of vertical and horizontal web reinforcement on the behavior of squat recycled concrete shear walls. Chen et al. [25] tested three recycled concrete shear walls, with 100% replacement ratio of RCA subjected to low-cyclic loading.

Based on the limited studies on the low rise RAC shear walls, it was confirmed that the RAC shear wall can be used in the seismic design. However, the studies considering the different Recycled Coarse Aggregates (RCA) or Recycled Fine Aggregates (RFA) replacement percentage are rare. In this study, a total of eight RAC shear walls with different RCA or RFA replacement percentages were designed and fabricated. The low cyclic loading tests were carried out on the eight specimens. The test parameters were the replacement percentage of RCA or RFA, reinforcement ratio, axial force ratio. In order to enhance the mechanical behavior of the RAC shear wall, among the eight specimens, one specimen was the strengthened RAC shear wall. In the strengthened RAC shear wall, the X-type rebars brace was encased, and the mechanical behavior was expected to increase significantly. The failure characteristics, hysteretic behavior, strength and deformation capacity, strain characteristics and stiffness of the eight wall specimens were studied.

## 2. Experimental Program

### 2.1. Mix Proportions

**Figure 1:**
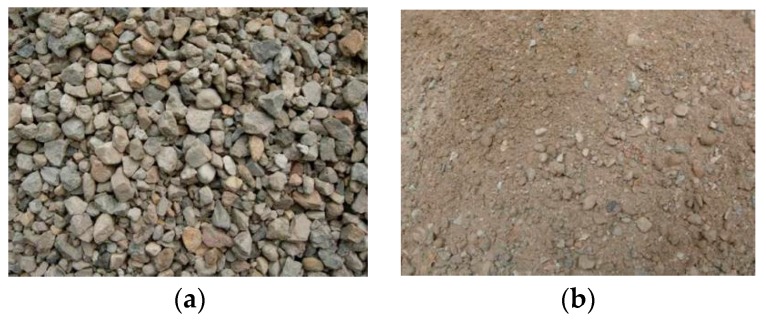
Recycled Aggregates. (**a**) Recycled Coarse Aggregates (RCA); (**b**) Recycled Fine Aggregates (RFA).

The waste concrete used in the experiment came from a concrete structure with over 20 years history in Beijing, China. The waste concrete was processed and sieved into the RCA and RFA. The recycled aggregates with particle size 5–25 mm were used as RCA, and the recycle aggregates with particle size 0.16–5 mm were used as RFA. The photographs of the RCA and RFA adopted in the experiment are shown in Figure 1a,b, respectively. 

**Figure 2:**
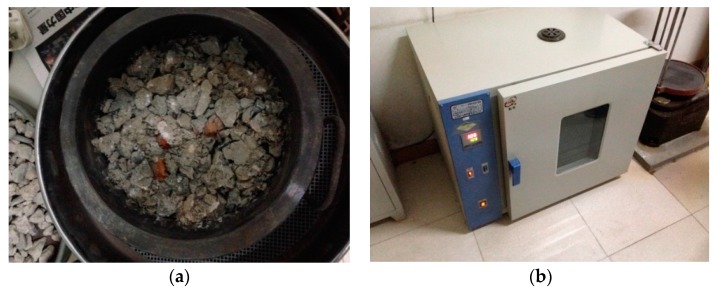
Instruments used for measuring the physical properties of the aggregates. (**a**) Crush index; (**b**) Water absorption.

**Figure 3:**
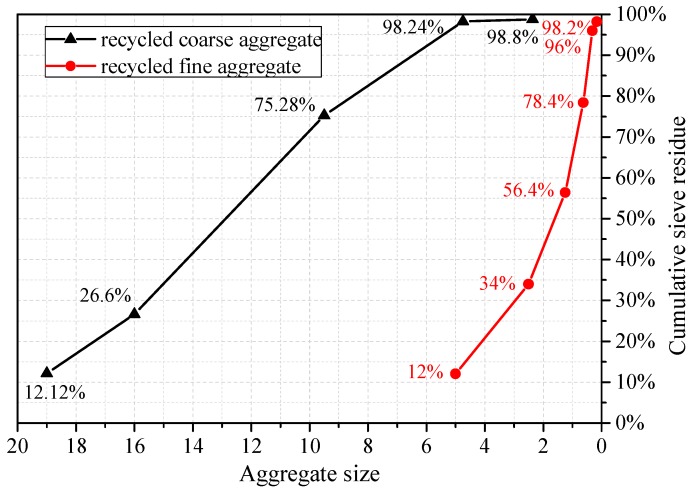
Particle size distribution of recycled coarse and fine aggregates.

The basic physical properties of RCA and RFA in accordance with the standards [26,27] are listed in Table 1. Some of the instruments used for measuring the physical properties of the aggregates are shown in Figure 2. The particle size distribution test results of RCA and RFA in accordance with the standards [26,27] are shown in Figure 3. Based on the principle of equal compressive strength of concrete, the mix proportions of the conventional and recycled concrete are shown in Table 2. Note that for the recycled aggregates, the water absorption is higher. So when the using of the recycled aggregates increases, more additional water was added. Portland cement P·I was used in all mixtures. The natural coarse and fine aggregates were from river. And the natural aggregates (both coarse and fine) are siliceous.

Three cubic specimens with 150 mm × 150 mm × l50 mm size were casted and tested in accordance with national standard [28] to obtain the cubic compressive strength *f_cu_*^150^ of the concrete. Note that the cubic compressive strength is different from the cylinder compressive strength. Cylinder compressive strength of concrete *f_c_*’ can be calculated based on Equation (1) [29].

(1) $$f_{c}^{’}=(0.66+0.002f_{cu}^{150})f_{cu}^{150}\geq 0.76f_{cu}^{150}$$

Six prism specimens with 150 mm × 150 mm × 300 mm were casted and tested in accordance with national standard [28] to obtain the Young’s modulus *E_c_* of the concrete. The mechanical properties of concrete and reinforcing bars in accordance with the national standard [30] are shown in Table 3 and Table 4, respectively.

### 2.2. Test Specimens

**Figure 4:**
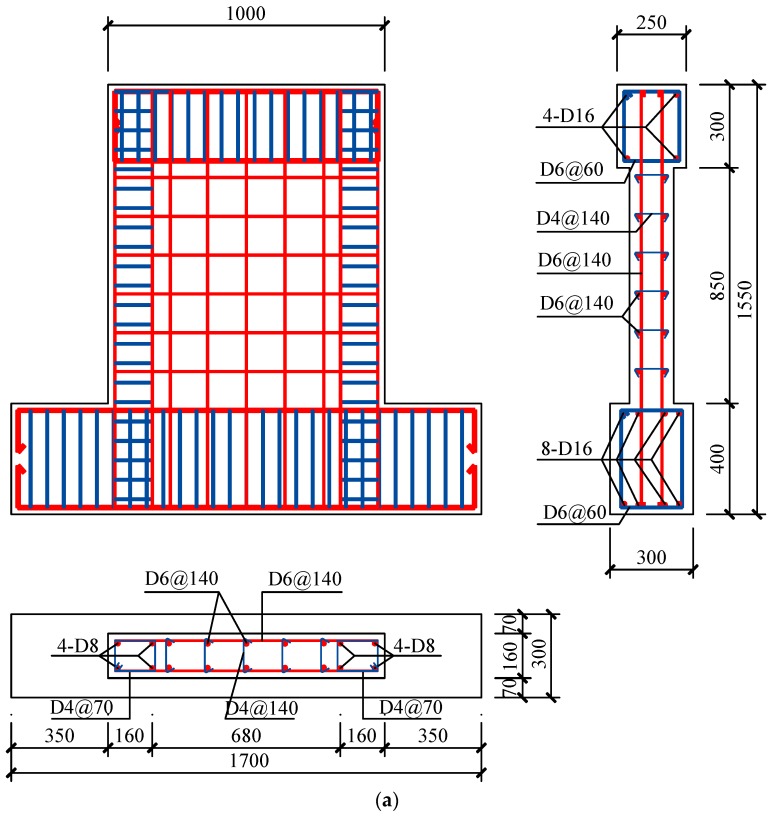

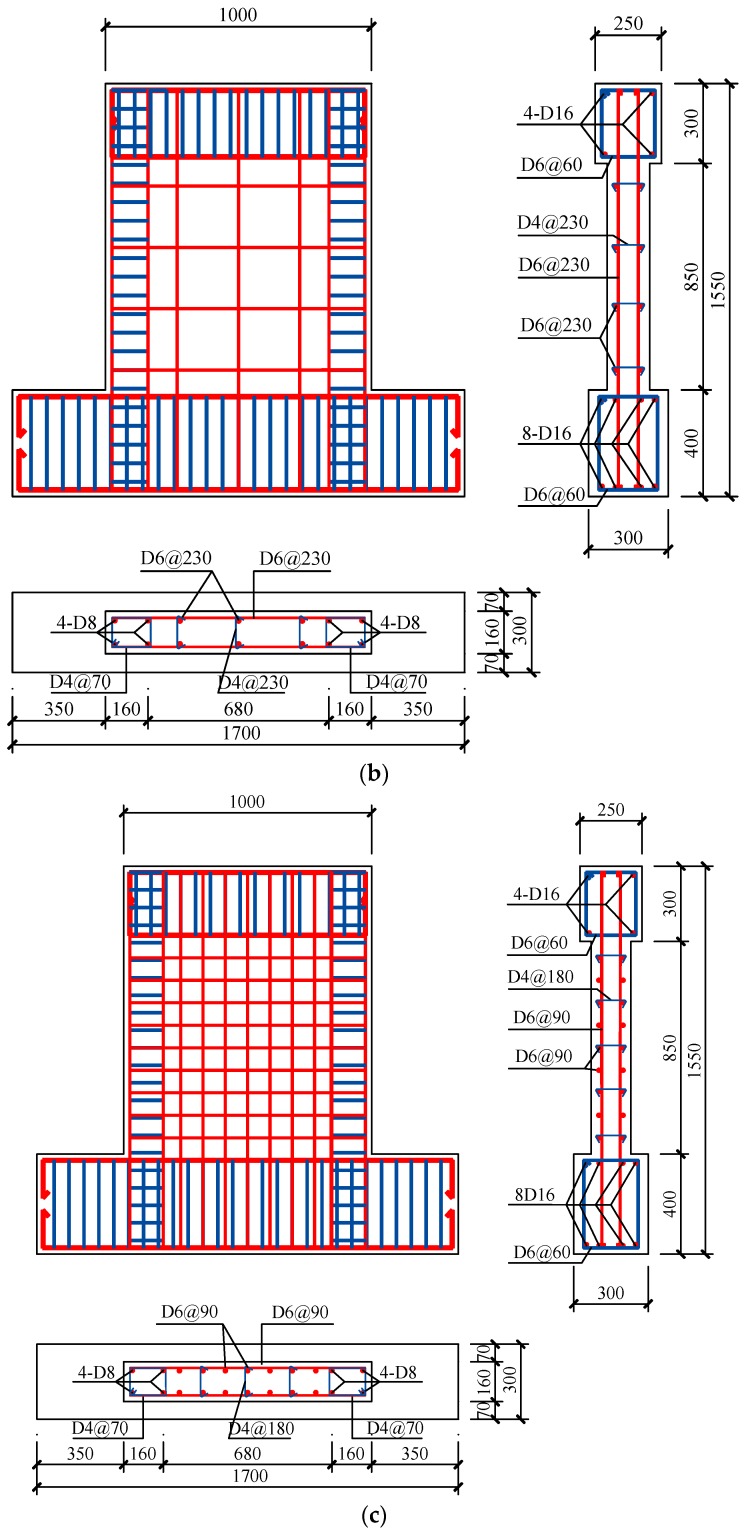

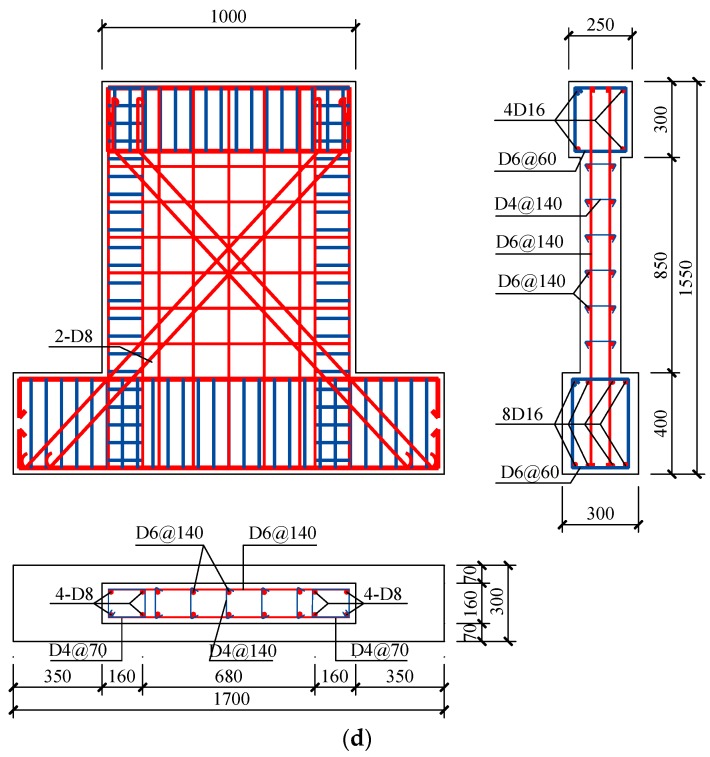
Specimen details. (**a**) Specimens W1, W2, W3, W4 and W5; (**b**) Specimen W6; (**c**) Specimen W7; (**d**) Specimen W8.

A total of eight rectangular shear walls were designed and fabricated. The specimens were numbered from W1 to W8. The design parameters of the specimens are listed in Table 5, the details of the specimens are shown in Figure 4. Specimen W1 used the conventional concrete with natural coarse and fine aggregates. Specimens W2 and W3 used the 50% and 100% RCA, respectively. Specimens W4, W5, W6, W7 and W8 used the 100% recycled coarse and fine aggregate. 

The overall size of the 8 concrete shear wall specimens was identical. The width of the wall was 1000 mm, and the height of the wall was also 1000 mm (from wall base to the loading point). The height-to-width ratio was 1.0. Reinforced concrete shear walls with a height-to-width ratio of less than 2.0 are commonly used in low rise buildings because of good performance in horizontal load resistance and drift control. Hence, the shear wall tested in this experiment was defined as the low rise shear wall. 

All the specimens had the boundary elements at both sides of the wall. The vertical reinforcing bars of the boundary elements were D8 (Diameter: 8 mm), while the stirrups at the boundary elements were D4@70 (Diameter: 4 mm, spacing: 70 mm). Reinforcing bars D6@140 (Diameter: 6 mm, spacing: 140 mm) were used for both vertical and horizontal web reinforcing bars of specimens W1, W2, W3, W4, W5 and W8. The reinforcement ratio was 0.25% for these specimens. For specimen W6, D6@230 (Diameter: 6 mm, spacing: 230 mm) were used for vertical and horizontal reinforcing bars, while for specimen W7, the reinforcing bars D6@90 (Diameter: 6 mm, spacing: 90 mm) were used. The reinforcement ratios of specimen W6 and W7 were 0.15% and 0.40%, respectively. Specimen W8 had the identical design variables with specimen W4, except that the additional X-shaped rebars brace (4-D8: four rebars with diameter of 8 mm) was encased in specimen W8. The encased X-shaped brace was expected to increase the strength and deformation capacity of the RAC shear wall. According to the fabrication of the specimen W8, the X-shaped brace could be easily encased in the shear wall specimen. 

Two kinds of axial force ratios, 0.15 and 0.30 were adopted for the test. The axial force ratio of the specimen W5 was 0.30, while, for other specimens, the axial force ratio was 0.15. In accordance with the JGJ 138-2012 provisions [31], the axial force ratio *n* is calculated as follows:

(2) $$n=N/(f_{c}^{’}A_{c}+f_{y}A_{s})$$

where, *N* is the axial force, *A_c_* is the sectional area of the concrete; *f_c_*’ is the cylinder compressive strength of the concrete; *f_y_* is the yield strength of the steel material; *A_s_* is the total sectional area of the steel material.

### 2.3. Test Setup and Measurements

**Figure 5:**
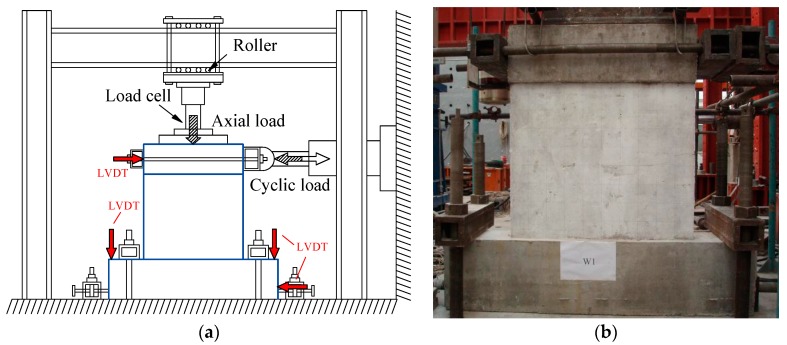
Test setup. (**a**) Loading scheme and measurements; (**b**) Photograph of loading.

**Figure 6:**
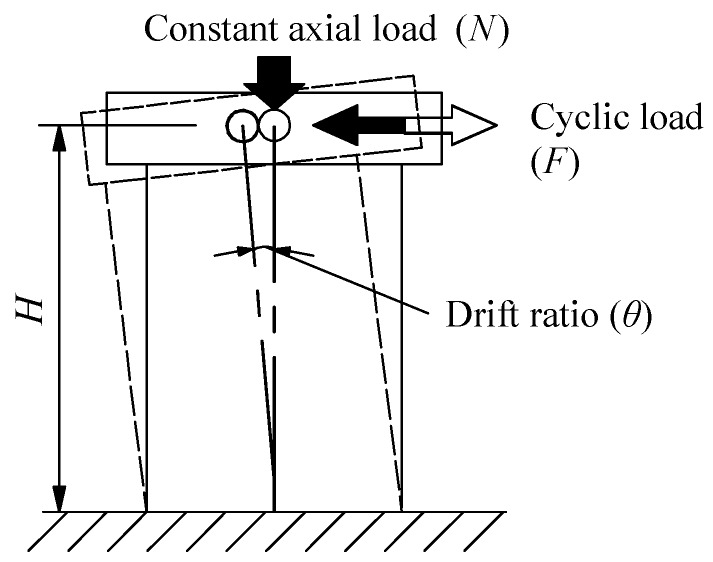
Definition of drift ratio *θ*.

Loading program: The test setup is shown in Figure 5. The foundation beam of the specimen was securely clamped to the reaction floor. The shear wall top was connected with a vertical and a horizontal hydraulic jack. The target vertical load was firstly subjected by vertical jack. After that, cyclic horizontal loads were applied quasi statically to the loading beam, and vertical load was kept constant through the entire test. The loading program was determined according to the Chinese specification: Testing Methods for Earthquake Resistant Building [32]. Before the yielding of the specimen, loading process was load-controlled mode. After the yielding of the specimen, the loading process was controlled by the horizontal displacement Δ. In the discussions later, the drift ratio *θ*, rather than the horizontal displacement Δ is used. It is defined as *θ* = Δ/*H*, where Δ is the horizontal displacement, and *H* is the height from the loading point to the base of wall, as shown in Figure 6.

Measurement: The loads, displacements and strains were measured during the test. Load cells were used to measure the vertical load and the horizontal load applied to the specimens. The Linear Variable Differential Transformers (LVDTs) were used to measure the displacement of the specimens. The layout of the LVDTs is shown in Figure 5a. Two LVDTs were used to measure the horizontal displacements at the loading beam, which was 1000 mm from surface of the foundation. Another two LVDTs were used on the foundation to monitor the slippage and lean of the foundation. Strain gauges were mounted at the vertical reinforcing bar of the boundary elements, the vertical and horizontal distributed reinforcing bars and the X-shaped rebars brace.

## 3. Test Results and Analysis

### 3.1. Failure Characteristics

**Figure 7:**
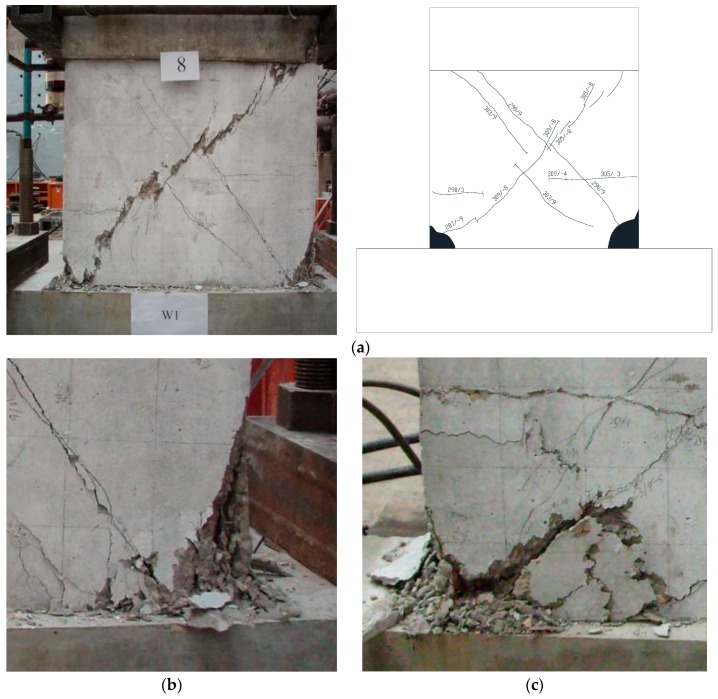

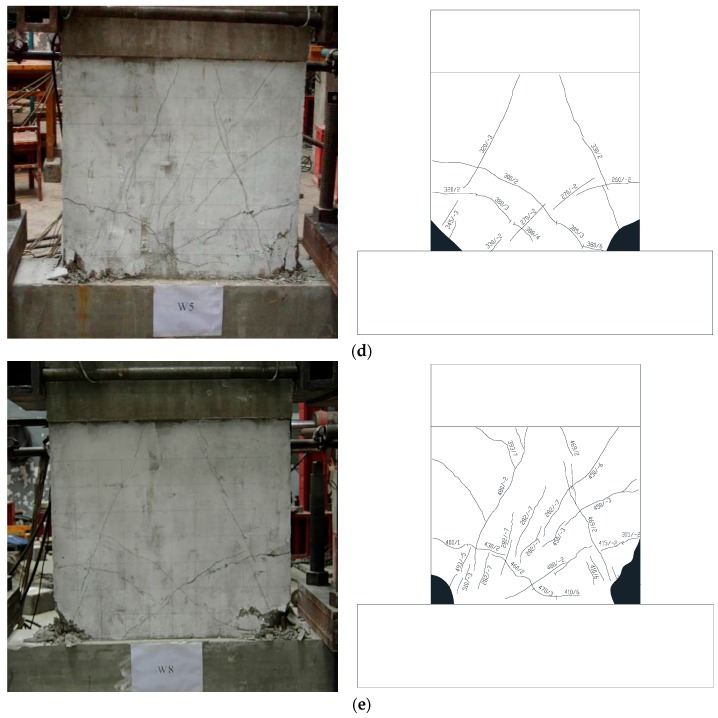
Failure characteristics. (**a**) Specimen W1; (**b**) Concrete crush of specimen W1; (**c**) Concrete crush of specimen W4; (**d**) Specimen W5; (**e**) Specimen W8.

Figure 7a shows the failure characteristics of specimen W1. The failure process of specimen W1 was as follows: when the horizontal load was 195 kN, the horizontal short cracks formed at the base of the boundary elements. With the process of the loading, the cracks developed into the web region obviously and the inclined cracks developed and climbed along the height of the wall. With the development of the loading, the crush of the concrete at the base of the wall became significant. Other specimens W2 and W3 with RCA had almost identical failure characteristics with specimen W1. However, for specimen W4 with 100% RCA and 100% RFA, the damage of the concrete at the base of the wall was more serious than specimen W1 with natural aggregates, as shown in Figure 7b,c, respectively. This indicated that more cumulative damages occurred for those specimens using the recycled aggregates. In order to prevent the serious damages, in the design of the RCA or RFA concrete shear walls, some strengthen method, such as the Fiber Reinforce Plastic (FRP) or steel plate strengthen method may be taken into account to reduce the concrete damages. 

Figure 7d shows the failure characteristics of the specimen W5 with higher axial force ratio. As shown, compared with the specimen W1, no obvious 45° inclined cracks were found due to the higher axial force ratio. Figure 7e shows the failure characteristics of the specimen W8 with the X-shaped brace. Different from specimens W1, W2, W3 and W4, no obvious 45° inclined cracks were found. It is confirmed that the brace had a positive effect on restraining the inclined cracks. Since the X-shaped brace is simple and economical during the design and construction of the concrete shear walls, when using RCA or RFA, it is suggested to install the X-shaped brace to reduce the damage of the concrete. 

### 3.2. Hysteretic Behavior and Strengths

**Figure 8:**
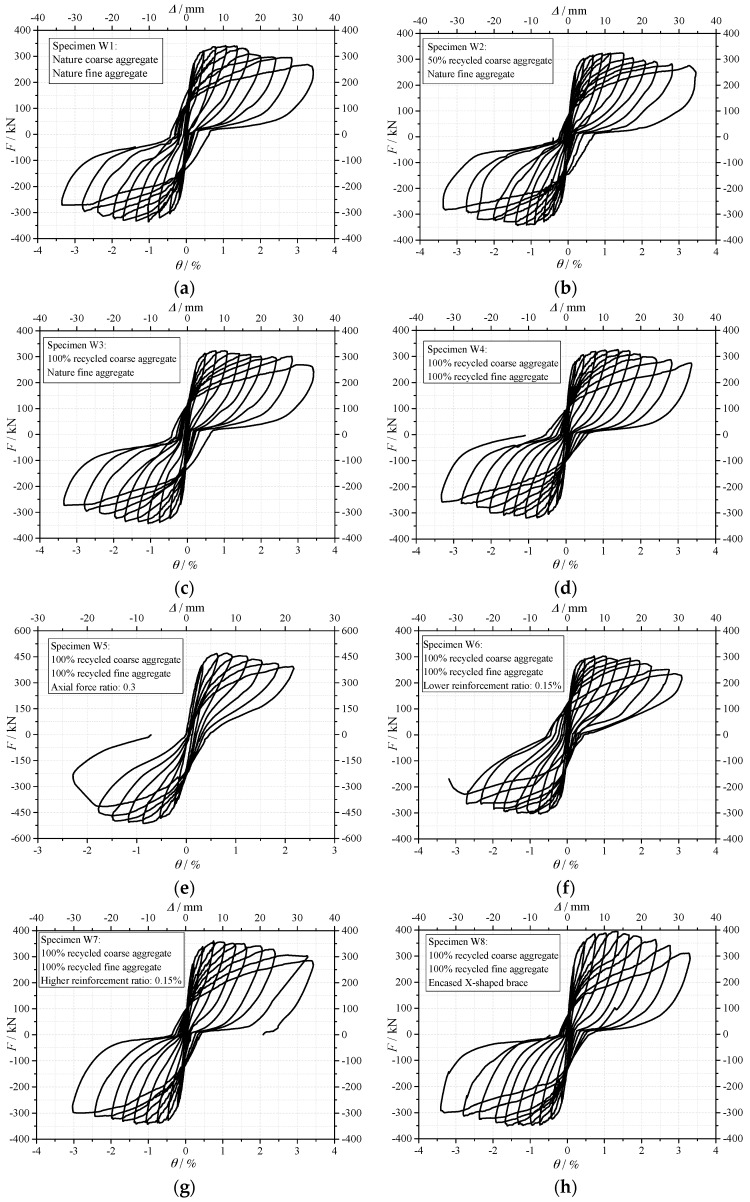
Hysteretic curves of the horizontal load *F*-drift ratio *θ.* (**a**) Specimen W1; (**b**) Specimen W2; (**c**) Specimen W3; (**d**) Specimen W4; (**e**) Specimen W5; (**f**) Specimen W6; (**g**) Specimen W7; (**h**) Specimen W8.

The “horizontal load *F*-drift ratio *θ*” hysteretic curves are shown in Figure 8. During the loading process, from the start of the loading to 1.0% (0.8% for specimen W5) drift ratio, the ductility behavior was good for all the specimens, no obvious strength degradation occurred. From 1.0% to 2.0% drift ratio, the strength of the specimens degraded gradually. Pinch phenomenon can be observed in the hysteretic curves of all specimens, especially for specimen W5 with higher axial force ratio. It is proved that the specimens with recycled aggregates can have the stable and plump hysteretic behavior. 

**Figure 9:**
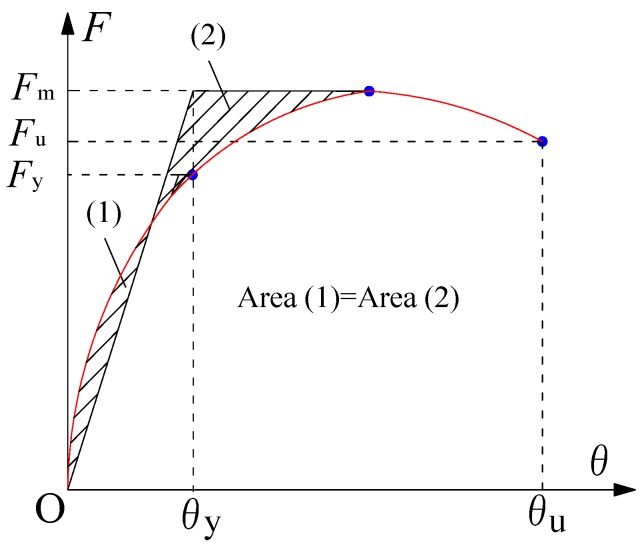
Definition of *F_y_*, *F_m_* and *F_u_*.

Table 6 shows the strength and deformation capacity of the specimens. In the table, *F_c_* is the crack load, *F_y_* is the yield load, *F_m_* is the maximum load, *θ_c_* is the crack drift ratio, *θ_y_* is the yield drift ratio and *θ_u_* is the ultimate drift ratio. Where, the *F_c_* and *θ_c_* was the load and drift ratio when the initial cracking occurred. The yield load *F_y_* and *θ_y_* was determined by the energy equivalent method [33], as shown in Figure 9. *θ_u_* is the ultimate drift ratio, which is defined as the strength decreases to 85% of the maximum load.

The parametrical studies are as follows:

(1) Influence of recycled aggregates

**Figure 10:**
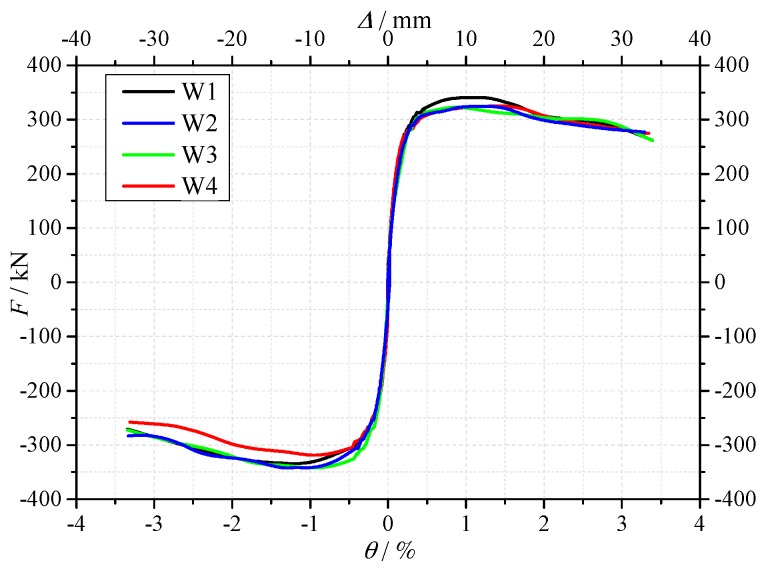
The skeleton curves of the specimens W1–W4.

Figure 10 shows the comparison of the skeleton curves of the specimen W1, W2, W3 and W4. As shown in Figure 10 and Table 6, the observations are as follows.

Influence of RCA: The skeleton curves were almost identical for specimen W1, W2 and W3. The crack load, yield load and maximum load were almost identical. It is confirmed that the using of the RCA and its replacement percentage had almost no influences on the strength of the shear walls.

Influence of RFA: The yield load and maximum load of specimen W4 were 2.6% and 3.1% lower than specimen W3, respectively. Considering the cubic compressive strength of the concrete used in specimen W4 was slight lower than that of specimen W3, it is confirmed that the RFA also had almost no influence on the strength of the shear wall.

From above, it is indicated that for the limited configurations considered in this study, in the strength design of the recycled aggregates concrete shear wall, the strength design method for the normal concrete shear wall can be also applicable for the recycled aggregates concrete shear wall. 

(2) Influence of axial force ratio

**Figure 11:**
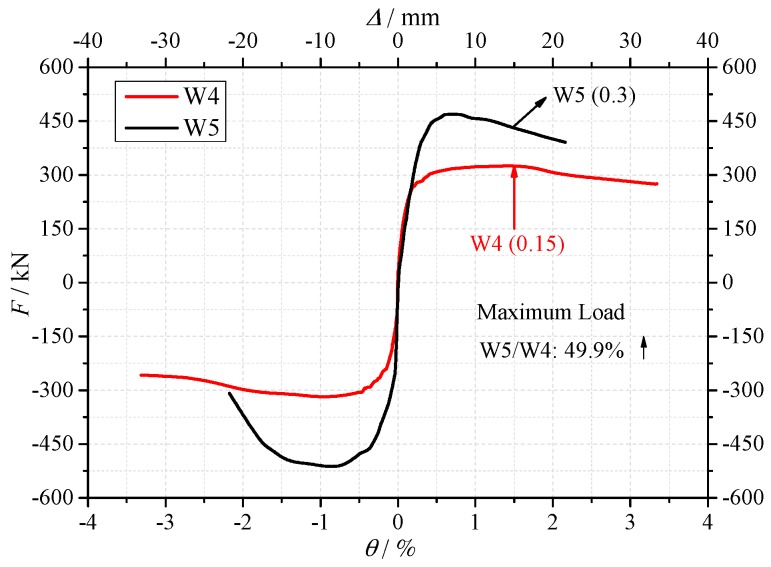
The skeleton curves of the specimens W4 and W5.

The axial force ratios of specimen W5 and specimen W4 were 0.3 and 0.15, respectively. The skeleton curves of the two specimens are shown in Figure 11. As shown in Figure 11 and Table 6, the crack load, yield load and maximum load of specimen W5 were 61.4%, 47.5% and 49.9% higher than specimen W4, respectively. It is indicated that under higher axial force ratio, the shear resistance of the shear wall increased significantly. This is mainly due to two reasons: (1) with the increase of the axial force, the shear force carried by the concrete wall increases; (2) The development inclined cracks will be restrained, in consequence, the shear resistance increases. 

(3) Influence of distributed web reinforcing bars

**Figure 12:**
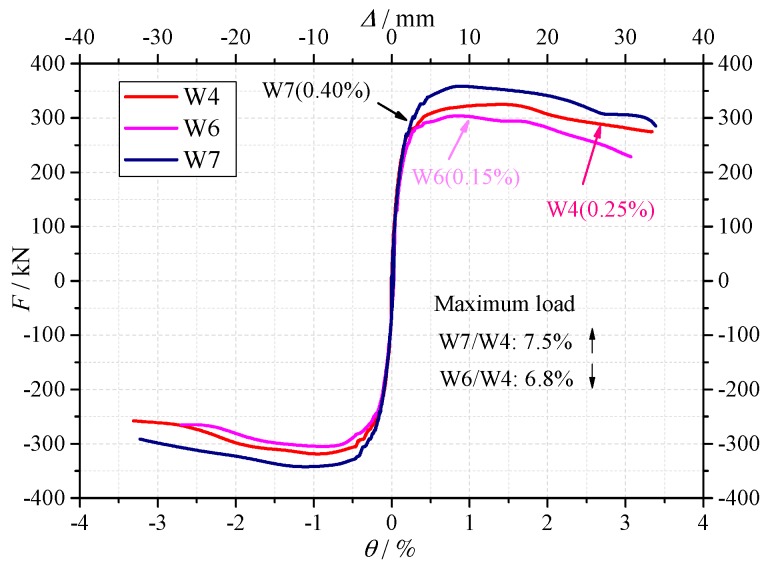
The skeleton curves of the specimens W4, W6 and W7.

The reinforcement ratios of W4, W6 and W7 were 0.25%, 0.15% and 0.40%, respectively. Figure 12 shows the skeleton curves of these three specimens. The reinforcement ratio had a certain influence on the strength of the shear walls. The crack load, yield load and maximum load of specimen W6 were 3.7%, 5.0% and 6.8% lower than specimen W4, respectively. While for specimen W7, the crack load, yield load and maximum load were 3.8%, 5.9% and 7.5% higher than specimen W4, respectively. In general, with the increasing of the reinforcing bars, the strength of the shear wall increased. It is confirmed that no matter the normal concrete or the recycled concrete are used in the shear wall, the reinforcement ratio is an important design factor. 

(4) Influence of encased X-shaped brace

**Figure 13:**
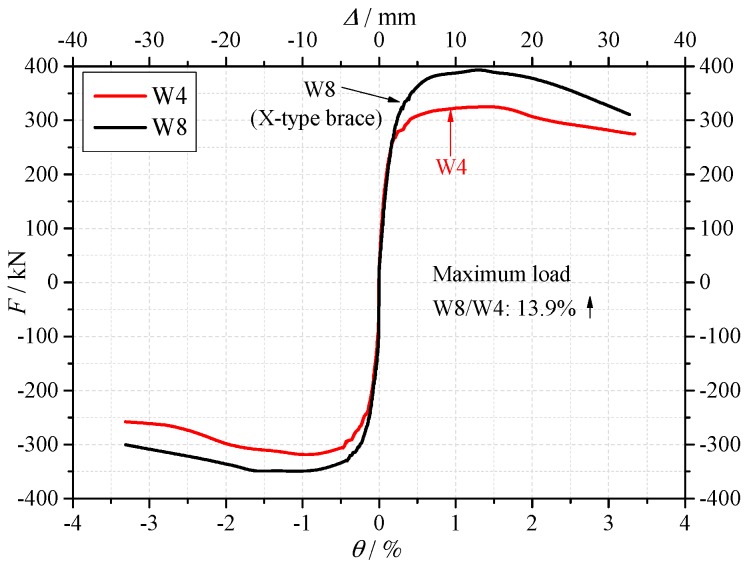
The skeleton curves of the specimens W4 and W8.

As shown in Figure 13, compared with specimen W4, specimen W8 was encased with the X-shaped rebars brace. It is confirmed that the crack load, yield load and maximum load increased by 3.9%, 11.5% and 13.9%, respectively. Even when compared specimen W8 with W7 which had a dense layout of the web reinforcing bars, the maximum load and yield load of specimen W8 were 6.0% and 5.0% higher than that of specimen W7, respectively. The encased X-shaped rebar brace was proved effective in enhancing the strength of the RAC shear wall. In the real engineering design and construction, the engineers always have a certain concern in using the RAC because of the mechanical behavior degradation of the RAC. By using the X-type rebars brace, which is low cost and simple in construction, the strength can increase significantly. The X-type rebars brace can be considered as an effective constructional measure to improve the application of the RAC shear wall. 

### 3.3. Ductility

**Figure 14:**
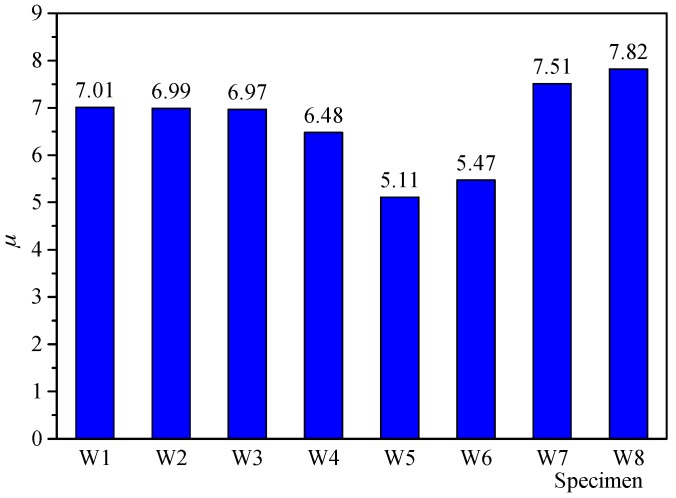
Ductility ratio *μ* of specimens.

Ductility ratio *μ* is an index to describe the deformation capacity of the shear walls without significant strength degradation. Ductility ratio *μ* is defined as *μ* = *θ_u_*/*θ_y_*. The higher the ductility ratio is, the better deformation capacity becomes. The ductility ratio of each specimen is shown in Figure 14. From Figure 14, according to the limited configurations considered in this study, the following observations can be obtained.

1. The RCA replacement percentages of the specimens W1, W2 and W3 were 0%, 50% and 100%, respectively. However, the ductility ratios of these specimens were 7.01, 6.99 and 6.97, respectively. Almost identical ductility ratios were confirmed. The using of the RCA and its replacement percentage had no influence on the ductility of the shear wall.
2. The specimens W4 and W3 differed in the RFA replacement percentage, the percentage was 0% for specimen W3 and 100% for specimen W4. The ductility ratio of specimen W4 was 7.0% lower than that of specimen W3. It is indicated that compared with RCA, the using of RFA had noticeable influence on the ductility of the shear wall. When using the RFA, the ductility degradation should be taken into consideration.
3. When comparing specimen W8 with W4, the ductility ratio increased by 20.7%. It is an indication that the encased X-shaped brace had an obvious effect on enhancing the ductility of the shear wall. Even comparing specimen W8 with W1 using conventional concrete, the ductility increased by 11.4%. In the seismic design of the RAC shear wall, the X-shaped rebar brace was an efficient method to improve the ductility of the RAC shear wall. Especially when using the RFA in the shear walls, the using of the X-shaped brace is recommended.
4. With the increase of the axial force ratio, the ductility decreased significantly. The ductility ratio of specimen W5 decreased by 22.5% compared with specimen W4. It is indicated that in the seismic design of the RAC shear wall, the axial force ratio should be seriously restricted.
5. The ductility was significantly affected by the reinforcement ratio of the wall. The ductility ratio of specimen W6 was 15.5% smaller than that of specimen W4, due to the smaller reinforcement ratio of the specimen W6. Meanwhile, the ductility ratio of specimen W7 was 15.9% higher than that of specimen W4.

Above all, it is concluded that although the using of the recycled concrete have almost no effect on the strength of the shear wall, the ductility of the wall will be reduced when using the RFA. When the shear wall needs to be designed with high deformation capacity, the using of the RFA is suggested to be restrained. If the RFA must be used in some situations, some other construction measures, such as the X-type bar or the high ratio reinforcement, are needed. 

### 3.4. Strain Characteristics

**Figure 15:**
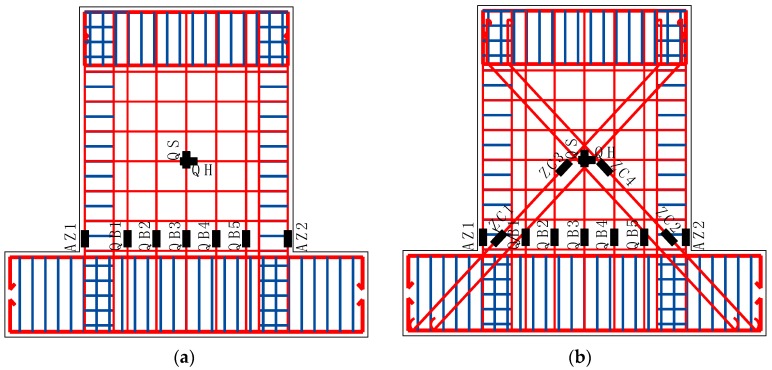
The location and numbering of the strain gauges. (**a**) Specimens W1, W2, W3, W4 and W5; (**b**) Specimen W8.

The location of the strain gauges are shown in Figure 15. Strain characteristics of half of the wall were studied because the symmetry of the strain gauges. As shown, the gauge at the vertical reinforcing bar of the boundary elements was named AZ1, the gauges at the vertical distributed reinforcing bar were QB1, QB2 and QB3. The gauges at the X-shaped rebars brace were ZC1 and ZC3. According to the results of the strain gauges, the vertical reinforcing bars were all confirmed yielding. The layout and design of the reinforcing bars were proved to be effective. Table 7 shows the values of the horizontal load and its loading cycle when the gauge reached the yielding strain *ε_y_* for specimen W4 and W8.

From Table 7, it is confirmed that for specimen W4, the yielding sequence of the strain gauges was AZ1, QB1, QB2 and QB3. For specimen W8, the yielding sequence of the strain gauges was AZ1, QB1, QB2, ZC1 and QB3. It is confirmed that the yielding of the reinforcing bars of specimen W8 was delayed compared with that of specimen W4, because the encased X-type rebars brace restricted the development of the inclined cracks. This indicates that the encased brace is able to carry part of the lateral force and ease the stress of the vertical reinforcing bars.

### 3.5. Degradation of Stiffness

#### 3.5.1. Degradation of Stiffness

**Figure 16:**
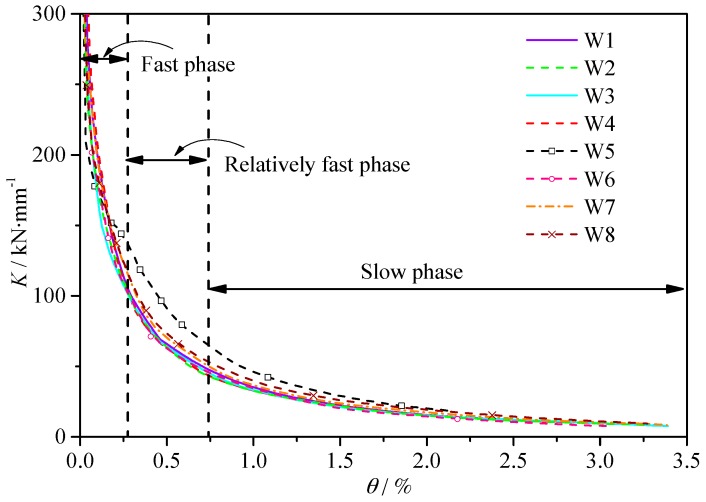
Degradation of stiffness.

Figure 16 shows the average secant stiffness at different drift ratio. The average secant stiffness of the test specimens is defined as:

(3) $$K_{i}=\frac{\left(F_{i}^{+}/\Delta_{i}^{+}+F_{i}^{-}/\Delta_{i}^{-}\right)}{2}$$

where, *K_i_* is the average stiffness of the minus and plus loadings of the *i^th^* cycle; *F_i_*^+^ is the maximum load of the plus loading of the *i^th^* cycle; *F_i_*^−^ is the maximum load of the minus loading of the *i^th^* cycle; Δ*_i_*^+^ is the maximum drift ratio of the plus loading of the *i^th^* cycle; Δ*_i_*^−^ is the maximum load of the minus loading of the *i^th^* cycle.

The stiffness of the shear wall became smaller with the increase of the drift ratio. In general, the degradation of stiffness can be roughly divided into three phases: (1) Fast degradation phase, which is from the start of the loading to the appearance of the crack; (2) Relatively fast degradation phase, which is from the appearance of the crack to the yielding of the specimens; (3) Slow degradation phase, which is from the yielding of the specimens to the end.

Table 8 shows the experimental results of the average secant stiffness of the specimens. Where, *K*_0_ is the initial stiffness, *K_c_* is the crack stiffness, *K_y_* is the yield stiffness, *β_co_* (=*K_c_*/*K*_0_) is the degradation of stiffness from the start of the loading to the crack point. *β_yo_* (=*K_y_*/*K*_0_) is the degradation of stiffness from the start of the loading to the yield point. *β_yc_* (=*K_y_*/*K_c_*) is the degradation of stiffness from the crack point to the yield point.

From Table 8 and Figure 16, it is confirmed that:

1. Despite the difference of the concrete type, reinforcement ratio and axial force ratio, the initial stiffness, were almost identical. Initial stiffness was mainly decided by the concrete strength and specimen dimensions.
2. When comparing specimens W2, W3 and W4 with specimen W1, the *K_c_*, *K_y_*, *β_co_*, *β_yo_* were found almost identical. Both the RCA and RFA were proved to have little effect on the stiffness and its degradation process of the shear wall.
3. As to specimen W8 with X-shaped brace, comparing with specimen W4, the *K_c_*, *K_y_*, *β_co_*, *β_yo_*, *β_yc_* increased 12.9%, 18.2%, 12.7%, 18.4% and 4.6%, respectively; Comparing with specimen W1 with conventional concrete, the *K_c_*, *K_y_*, *β_co_*, *β_yo_* and *β_yc_* increased 5.0%, 14.0%, 7.8%, 17.3% and 9.4%, respectively. The using of encased X-shaped rebars brace can significantly slow down the degradation of stiffness.

#### 3.5.2. Calculation of Initial Stiffness

**Figure 17:**
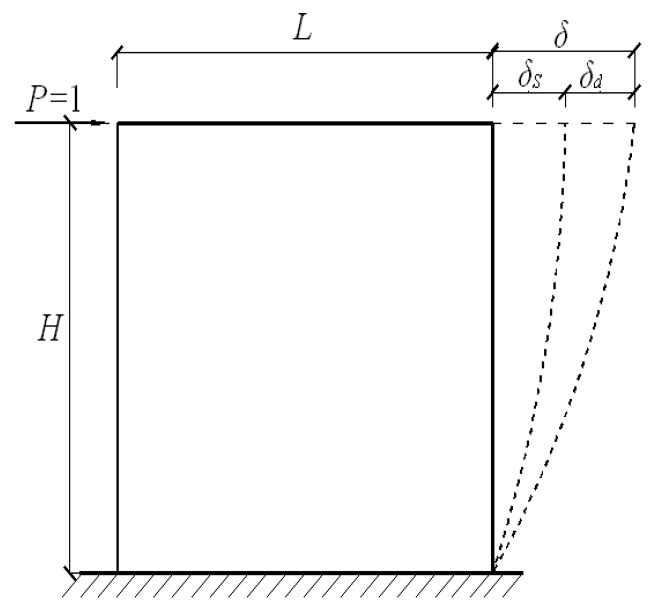
Calculation model of initial stiffness.

The calculation model of initial stiffness is shown in Figure 17. In Figure 17, *H* is the calculation height of the shear wall, *L* is the width of the shear wall, *δ_s_* is the shear deformation with unit-load. *δ_b_* is the bending deformation with unit-load. The total deformation with unit-load was *δ* = *δ_s_* + *δ_b_*. 

Hence, the initial stiffness of the wall was determined by the following calculation:

(4) $$K_{o}=\frac{1}{\delta_{s}+\delta_{b}}=\frac{1}{\frac{\mu H}{AG}+\frac{H^{3}}{3EI}}$$

where, *μ* is the shear stress distribution coefficient, *μ* = 1.2 for rectangular section; *A* is the total sectional area of the wall, *A* = *A*_0_ + *A*_1_, *A*_0_ is the net horizontal sectional area of the wall concrete, *A*_1_ is the sectional area by transforming the reinforcing bars to the concrete; *E* is the Young’s modulus of the concrete; *G* is the shear modulus of the material, *G* = 0.4*E*; *I* is the sectional moment of inertial of the wall.

Based on Equation (4), the following equations can be obtained.

(5) $$\delta_{s}=\frac{1.2H}{AG}=\frac{1.2H}{0.4AE}=\frac{3H}{AE}$$

(6) $$\delta_{b}=\frac{H^{3}}{3EI}$$

From Equations (4)–(6),

(7) $$K_{o}=\frac{1}{\delta_{s}+\delta_{b}}=\frac{1}{\frac{3H}{AE}+\frac{H^{3}}{3EI}}$$

The calculated initial stiffness of the shear walls based on the Equation (7) is shown in Table 9. The comparisons between experimental and calculated initial stiffness are also listed. The calculated results were in good agreement with the experimental results.

## 4. Discussions on Strength Evaluation of the Shear Walls

Two strength evaluation methods of the ACI318-14 [34] and EC8 [35] were adopted for evaluating the experimental strengths. For both methods, the concrete contribution and the web reinforcing bars contribution were taken into consideration. Furthermore, for specimen W8 with encased X-type rebars brace, the horizontal and vertical components of the brace were also taken into consideration in the calculation. The semi-empirical formula presented in the ACI 318-14, which ignores the effect of web vertical reinforcing bras and only accounts for the amount of horizontal reinforcing bars, provides the minimum requirements for the seismic design of RC shear walls. The strength design formula in the European code EC8, accounts for both vertical and horizontal web reinforcing bars and ignores concrete contribution for low rise walls subjected to low axial stresses. The strength evaluations in ACI 318-14 are as follow:

(8) $$V_{cal1}=A_{cv}(\alpha_{c}\sqrt{f_{c}^{’}}+\rho_{h}f_{yh})\leq 0.83A_{cv}\sqrt{f_{c}^{’}}$$

where, $\alpha_{c}$ is an aspect ratio dependent coefficient, which is 0.25 when $\frac{H_{w}}{l_{w}}\leq 1.5$, 0.17 when $\frac{H_{w}}{l_{w}}\geq 2$, and varies linearly between 0.25 and 0.17 when $\frac{H_{w}}{l_{w}}$ between 1.5 and 2.0. $H_{w}$ is the wall height, *l_w_* is the wall length, *A_cv_* is the gross area of concrete section bounded by web thickness and length of section in the direction of shear force considered in the case of walls, *ρ_h_* and *ρ_v_* are the horizontal web reinforcement ratio and the vertical web reinforcement ratio, respectively, *f_yh_* and *f_yv_* are the yield stress of web horizontal reinforcing bars and vertical reinforcing bars.

The equations of EC8 are as follows:

(9) $$V_{cal2}=[\rho_{h}f_{yh}(\frac{M_{n}}{V_{n}l_{w}}-0.3)+\rho_{v}f_{yv}(1.3-\frac{M_{n}}{V_{n}l_{w}})]t_{w}d_{w}\;if\;\frac{1.5P_{n}}{A_{cv}f_{c}^{’}}<0.1$$

(10) $$V_{cal2}=0.15t_{w}d_{w}\sqrt{f_{c}^{’}}+[\rho_{h}f_{yh}(\frac{M_{n}}{V_{n}l_{w}}-0.3)+\rho_{v}f_{yv}(1.3-\frac{M_{n}}{V_{n}l_{w}})]t_{w}d_{w}\;if\;\frac{1.5P_{n}}{A_{cv}f_{c}^{’}}>0.1$$

where *M_n_* is the moment at the wall bottom, *P_n_* is the axial loading, *V_n_* is the horizontal loading, *t_w_* is the wall thickness, *d_w_* is the distance from extreme compression fibre to location of resultant of forces in vertical reinforcing bars in tension.

**Figure 18:**
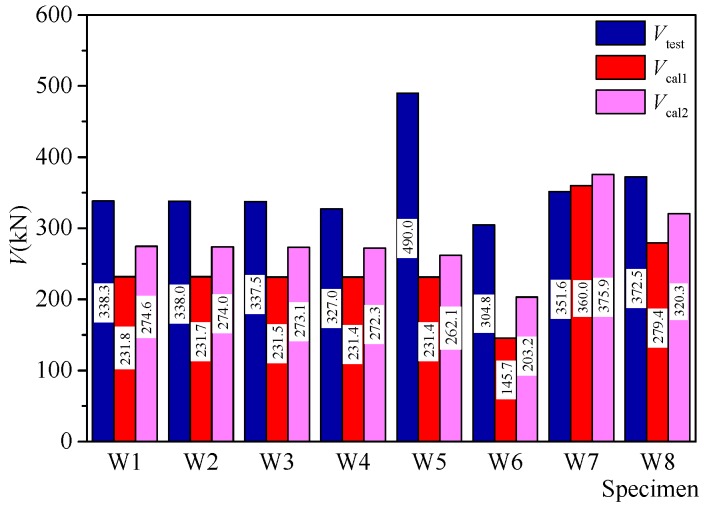
Experimental and calculated shear strengths.

Figure 18 shows the test results and the calculated results based on the two design codes. It is indicated that the evaluation based on the ACI 318-14 was more conservatively, while the EC8 strength evaluation method showed a better accuracy. The average ratio of the test value *V_test_* to the calculated value *V_cal_*_1_ was 1.54, and the average ratio of the test value *V_test_* to the calculated value *V_cal_*_2_ was 1.30. However, the comparison results of specimen W5 with higher axial force ratio had a higher error. This is because the two design codes consider few on the effect of axial stress.

## 5. Conclusions

In this study, low rise concrete shear walls using recycled coarse and fine aggregates concrete were designed and tested under low cyclic loading. According to the limited configurations considered in this study, the main conclusions are:

(1) The shear wall specimen fractured in shear domain mode, characterized by the diagonal cracks and crushing and spalling of the concrete.
(2) The using of the RCA had almost no influence on strength, ductility and stiffness of the shear walls.
(3) The using of the RFA had no obvious influence on the strength and stiffness of the shear walls, but the ductility decreased by 7.0% compared with the RFA shear walls.
(4) The reinforcement ratio showed a significant influence on the strength and ductility of the shear wall. With the increase of the reinforcement ratio, the strength and ductility increased.
(5) When the axial force ratio increased, the ultimate strength increased by 49.8%, but the ductility decreased by 22.5%. The axial force ratio should be restricted when using recycled aggregates in the low rise shear walls.
(6) When the X-shaped rebars brace was encased in the shear wall, the maximum strength and ductility ratio increased by 13.9% and 20.7%, respectively. When using recycled aggregates in the RC shear wall, encasing the X-shaped rebars brace can be considered as an effective and simple constructional measure. It is as an effective way to improve the application of the RAC shear wall.
(7) The shear strengths were calculated according to the ACI 318-14 and EC8. It is confirmed that the evaluation based on ACI 318-14 was more conservative, the EC8 formula showed a better accuracy.

## Figures and Tables

**Table 1:** Basic physical properties of RCA and RFA.

Recycled Aggregates	Crush Index (%)	Bulk Density (kg/m^3^)	Apparent Density (kg/m^3^)	Water Absorption (%)	Slit Content (%)
RCA	9.3	1253	2565	4.2	3.0
RFA	11.4	1307	2455	8.5	3.2

**Table 2:** Mix proportions of the concrete.

Group	Cement (kg/m^3^)	Water (kg/m^3^)	Additional Water (kg/m^3^)	NCA (kg/m^3^)	RCA (kg/m^3^)	NFA (kg/m^3^)	RFA (kg/m^3^)
RCA = 0%, RFA = 0%	406	195	0	1086	0	636	0
RCA = 50%, RFA = 0%	406	218	23	543	543	636	0
RCA = 100%, RFA = 0%	406	241	46	0	1086	636	0
RCA = 100%, RFA = 100%	406	295	100	0	1086	0	636

Note: NCA = Natural Coarse Aggregates, NFA = Natural Fine Aggregates.

**Table 3:** Mechanical properties of concrete.

Concrete Grade	RCA (%)	RFA (%)	*f_cu_*^150^ (N/mm^2^)	*f_c_*’ (N/mm^2^)	*f_cu_*^150^ (N/mm^2^)	*f_c_*’ (N/mm^2^)	*E_c_* (N/mm^2^)
			28 Days		at Test Days		
C30	0%	0%	34.84 (33.82)	26.48 (25.70)	36.45 (35.87)	27.70 (27.26)	3.13 × 10^4^
RAC30-1	100%	100%	31.97 (32.14)	24.30 (24.43)	34.82 (35.41)	26.46 (26.91)	2.35 × 10^4^
RAC30-2	50%	0%	33.57 (33.48)	25.51 (25.44)	36.02 (35.58)	27.38 (27.04)	2.60 × 10^4^
RAC30-3	100%	0%	33.13 (32.58)	25.18 (24.76)	35.39 (34.88)	26.90 (26.50)	2.74 × 10^4^

Note: 1. *f_cu_*^150^ is the cubic compressive strength, *f_c_*’ is the cylinder compressive strength; 2. The values in the bracket ( ) are the median values; 3. The test days for C30, RAC30-1, RAC30-2 and RAC30-3 are 52 d, 63 d, 55 d, 57 d, respectively.

**Table 4:** Mechanical properties of reinforcing bars.

Reinforcing Bar	Diameter (mm)	Yield Strength (N/mm^2^)	Maximum Strength (N/mm^2^)	Young’s Modulus (N/mm^2^)
D6	6	535.82	590.64	1.77 × 10^5^
D8	8	338.20	492.88	1.98 × 10^5^
D10	10	427.80	527.12	1.71 × 10^5^

**Table 5:** Specimen details.

Specimen	RCA Percentage	RFA Percentage	Reinforcement Ratio	Axial Force Ratio
W1	0	0	0.25%	0.15
W2	50%	0	0.25%	0.15
W3	100%	0	0.25%	0.15
W4	100%	100%	0.25%	0.15
W5	100%	100%	0.25%	0.30
W6	100%	100%	0.15%	0.15
W7	100%	100%	0.40%	0.15
W8	100%	100%	0.25% + brace	0.15

**Table 6:** Strength and deformation capacity of specimens.

Specimen	Crack Point		Yield Point		Maximum Load *F_m_* (kN)	Ultimate Drift *θ_u_* (%)
	Load *F_c_* (kN)	Drift *θ_c_* (%)	Load *F_y_* (kN)	Drift *θ_y_* (%)		
W1	134.5	0.06	308.5	0.417	338.3	2.925
W2	132.2	0.063	305.9	0.415	338.0	2.896
W3	131.0	0.061	308.4	0.408	337.5	2.843
W4	132.5	0.064	300.5	0.421	327.0	2.728
W5	213.9	0.131	443.2	0.374	490.0	1.908
W6	127.7	0.070	285.4	0.419	304.8	2.291
W7	137.5	0.059	318.3	0.398	351.6	2.988
W8	136.5	0.058	335.0	0.397	372.5	3.100

**Table 7:** Horizontal loads and cycle number of loading when reaching yield strain.

Location Number of Strain Gauges		AZ1	QB1	QB2	QB3	ZC1
Specimen W4	Horizontal load	260.30	266.46	271.15	306.61	No brace
	Number of loading cycles	+1	+1	+2	+2	
Specimen W8	Horizontal load	+263.25	+233.29	+303.39	+309.79	+347.89
	Number of loading cycles	+1	+3	+3	+4	+3

**Table 8:** Experimental values of stiffness.

Specimen	*K_o_* (kN/mm)	*K_c_* (kN/mm)	*K_y_* (kN/mm)	*β_co_*	*β_yc_*	*β_yo_*
W1	794.14	224.17	73.98	0.282	0.330	0.093
W2	785.21	209.84	73.81	0.267	0.352	0.094
W3	781.97	214.80	75.60	0.275	0.352	0.097
W4	773.25	207.03	71.37	0.268	0.345	0.092
W5	772.25	163.27	118.67	0.211	0.727	0.153
W6	771.30	193.44	68.19	0.251	0.352	0.088
W7	774.66	231.43	79.97	0.299	0.346	0.103
W8	789.88	233.81	84.48	0.296	0.361	0.107

**Table 9:** Experimental and calculated results of initial stiffness.

Specimen	Experimental Results *K_o,exp_* (kN/mm)	Calculated Results *K_o,cal_* (kN/mm)	*K_o,exp_*/*K_o,cal_*
W1	794.14	776.18	1.02
W2	785.21	771.54	1.02
W3	781.97	768.45	1.02
W4	773.25	759.51	1.02
W5	772.25	757.45	1.02
W6	771.30	760.54	1.01
W7	774.66	762.54	1.02
W8	789.88	780.45	1.01

## Abbreviations

The following abbreviations are used in this manuscript:

RAC	Recycled Aggregates Concrete
RFA	Recycled Fine Aggregates
RCA	Recycled Concrete Aggregate
NAC	Natural Aggregates Concrete
RC	Reinforced Concrete
NCA	Natural Coarse Aggregates
NFA	Natural Fine Aggregates
